# Novel 3D embryo implantation model within macroporous alginate scaffolds

**DOI:** 10.1186/s13036-020-00240-7

**Published:** 2020-06-30

**Authors:** Dganit Stern-Tal, Hanna Achache, Liora Jacobs Catane, Reuven Reich, Tali Tavor Re’em

**Affiliations:** 1 School of Pharmacy, Institute for Drug Research, The Hebrew University of Jerusalem, 91120 Jerusalem, Israel; 2 Department of Pharmaceutical Engineering, Azrieli College of Engineering Jerusalem, 26 Yaakov Shreibom Street, 9103501 Jerusalem, Israel

**Keywords:** Embryo implantation, Alginate, Porous scaffold, Endometrium, Epithelial endometrial cells, Recurrent implantation failure, RIF, 3D in vitro model, Menstrual cycle, Hormone treatment

## Abstract

**Background:**

Implantation failure remains an unsolved obstacle in reproductive medicine. Previous studies have indicated that estrogen responsiveness, specifically by estrogen receptor alpha (ERα), is crucial for proper implantation. There is an utmost need for a reliable in vitro model that mimics the events in the uterine wall during the implantation process for studying the regulatory mechanisms governing the process. The current two-dimensional and hydrogel-based in vitro models provide only short-term endometrial cell culture with partial functionality.

**Results:**

Endometrial biopsies showed an increase in E-cadherin expression on the typical window of implantation of fertile women, compared to negligible expression in recurrent implantation failure (RIF) patients. These clinical results indicated E-cadherin as a marker for receptivity. Three-dimensional (3D) macroporous alginate scaffolds were the base for epithelial endometrial cell-seeding and long-term culture under hormone treatment that mimicked a typical menstrual cycle. The RL95–2 epithelial cell culture in macroporous scaffolds was viable for 3 weeks and showed increased E-cadherin levels in response to estrogen. Human choriocarcinoma (JAR) spheroids were used as embryo models, seeded onto cell constructs and successfully adhered to the RL95–2 cell culture. Moreover, a second model of HEC-1A with low ERα levels, showed lower E-cadherin expression and no JAR attachment. E-cadherin expression and JAR attachment were recovered in HEC-1A cells that were transfected with ERα plasmid.

**Conclusions:**

We present a novel model that enables culturing endometrial cells on a 3D matrix for 3 weeks under hormonal treatment. It confirmed the importance of ERα function and E-cadherin for proper implantation. This platform may serve to elucidate the regulatory mechanisms controlling the implantation process, and for screening and evaluating potential novel therapeutic strategies for RIF.

## Background

Embryo implantation is considered relatively ineffective in humans [1, 2], as the probability of implantation is only 25% per menstrual cycle [3]. Implantation rates are even lower in women who suffer from recurrent implantation failures (RIF) after a number of in vitro fertilization treatments [4]. It has been estimated that inadequate uterine receptivity is responsible for approximately two-thirds of the cases [5]. Previous studies have indicated that estrogen responsiveness, specifically by estrogen receptor alpha (ERα), is crucial for proper implantation [6]. Moreover, RIF patients have shown increased levels of Slug [4], an E-cadherin gene suppressor, relative to fertile women [6]. These studies suggest a positive correlation between ERα and E-cadherin and their role in the embryo implantation process. In this study, E-cadherin protein expression was assessed in RIF endometrium tissue and showed significantly lower protein levels relative to fertile women on day 21, the typical window of implantation (WOI).

Further examination of the regulatory mechanisms governing the complex embryo implantation process requires an in vitro model for uterine functionality; particularly since human experiments are ethically impractical and animal in vivo models suffer from major dissimilarities regarding their reproductive physiology and implantation rates compared to human. Constructing such a model requires either human endometrial primary cells, derived from Pipelle® sampling, or modified endometrial cell lines such as RL95–2, HEC-1A, Ishikawa, etc., [7] along with an embryo-like model such as human choriocarcinoma (JAR) spheroids [7]. Furthermore, estrogen and progesterone are also required for the endometrial culture since they are key hormones that regulate the menstrual cycle and are involved in the implantation process [8].

To date, most of the current in vitro implantation studies have been conducted on monolayer, two dimensional (2D) culture systems [9–13]. 2D limitations were addressed by cell seeding onto different coated substrates, such as Matrigel® [14, 15], and using advanced microfluidic systems [16]. However, 2D cell cultures, even when seeded onto thick gels, do not mimic the actual complex three-dimensional (3D) tissue, nor do they survive for more than a few days under hormonal treatment. Consequently, monolayer cultures cannot model the complex embryo implantation process, particularly the steps of apposition and invasion into the deeper layers of the endometrial tissue. To date, endometrial cells have been cultured in a number of 3D hydrogel systems, e.g., Matrigel® [17], collagen-Matrigel® composites [18], fibrin-agarose [19, 20] and gelatin [21]. These 3D hydrogel culture systems support a variety of physiological processes, such as cell–cell and cell–matrix interactions, and expression of well-characterized cellular biomarkers, indicating that cells cultured in a 3D environment can represent in vivo cellular behavior [19]. These hydrogel systems were advantageous compared to monolayer cultures; however, they were characterized by relatively short cultivation periods (hours to days) due to limited access to medium and paracrine factors, both crucial to fully represent the temporally dynamic endometrium tissue. No current in vitro uterine model emulates the entire periodic behavior and tissue functionality in terms of temporal embryo attachment at a specific time – the WOI.

This study presents a porous scaffold as an alternative culture method for enabling 3-week long culture of epithelial cells under hormone treatment that mimics the typical menstrual cycle. Porous 3D matrices are beneficial for long-term cell culture, drug delivery and cell transplantation [22–28], due to high mass transfer efficiency and better exchange of nutrients, oxygen, waste, etc. [29]. Macroporous alginate scaffolds have been extensively used for long-term primary human cultures due to their high porosity and interconnected pore structure [22–25]. These scaffolds have been thoroughly characterized by Cohen and colleagues, e.g., scaffold porosity, pore architecture and compressibility [29, 30], and cell viability, morphology and function [22, 24, 25, 31].

Macroporous alginate scaffolds were used for engineering endometrial models to mimic receptive vs. non-receptive tissue, using appropriate human epithelial endometrial cell lines, RL95–2 and HEC-1A, respectively. We constructed a 3D in vitro endometrial culture that was grown for 3 weeks, the length of the menstrual cycle, under hormonal conditions that mimic in vivo conditions. This model functioned as a novel implantation model that allowed us to examine the roles of ERα and E-cadherin throughout the menstrual cycle. JAR spheroid attachment served as a model for embryo implantation and to examine potential therapeutic solutions for RIF patients.

## Results

### E-cadherin expression in fertile women and in RIF patients

**Fig. 1:**
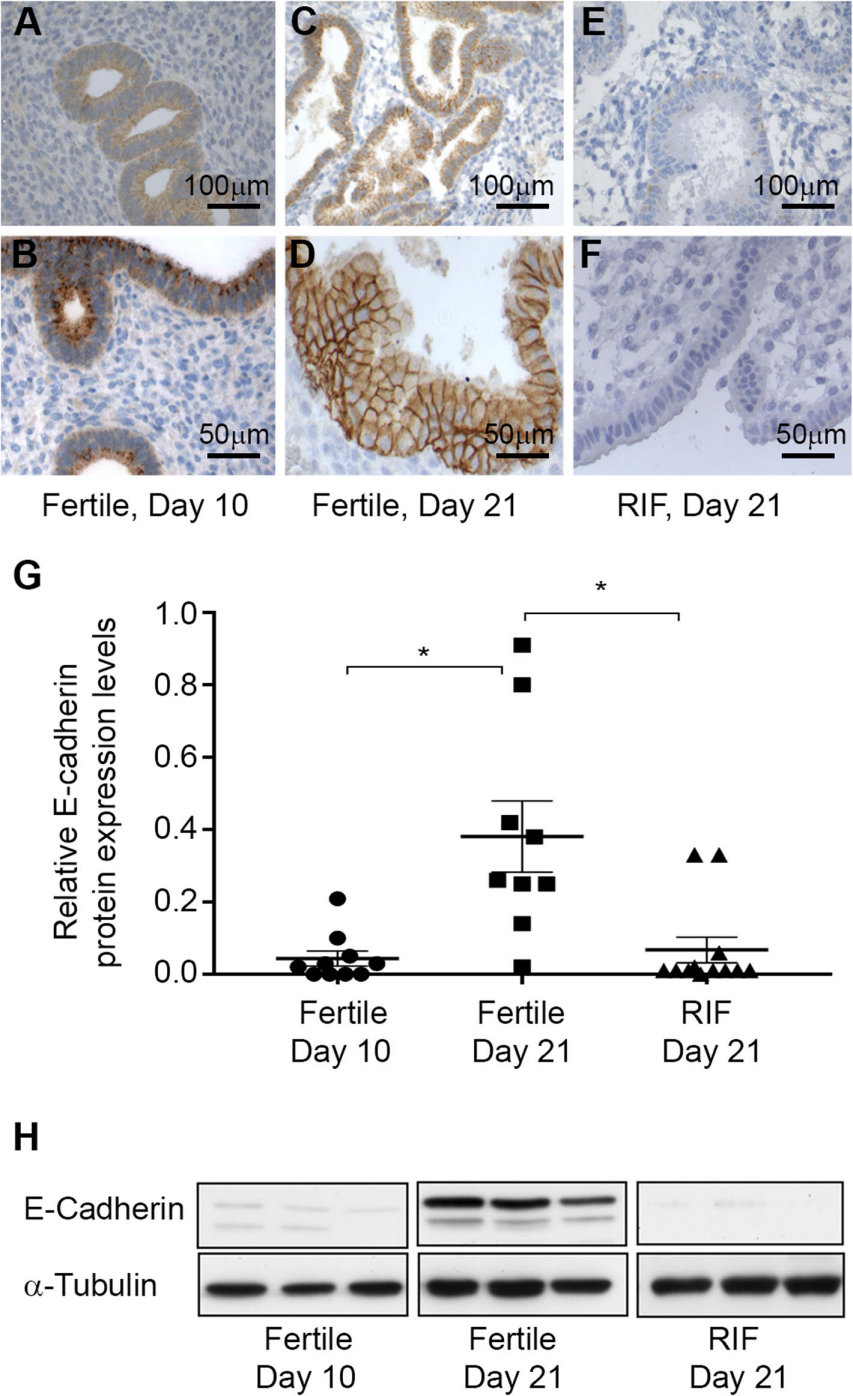
**A**-**F** E-cadherin expression in the endometrium of fertile and RIF patients. Representative photomicrographs of E-cadherin immunostaining of paraffin sections of fertile female endometrium (**A**, **B**) on day 10, and (**C**, **D**) day 21 when E-cadherin was localized in the epithelial cell membrane. Almost no E-cadherin expression was detected in RIF patients’ endometrial tissue on day 21 (**E**, **F**). (Bar: **A**, **C**, **E**: 100 μm; **B**, **D**, **F**: 50 μm). **G** Quantification of E-cadherin protein expression levels by Western blot (WB) of endometrium from fertile women on day 10 (*n* = 9) and day 21 of the menstrual cycle (*n* = 10), and of RIF patients on day 21 of the cycle (*n* = 12, *p* < 0.05, 1-way ANOVA, Dunnett’s post-test). Expression levels were quantified by the band intensity relative to α-tubulin. **H** Representative E-cadherin WB

E-cadherin immuno-staining of endometrium tissue from day 10 of the normal menstrual cycle of fertile women showed positive staining of E-cadherin in the epithelium (Fig. 1A and B). On day 21 of the menstrual cycle, the epithelium expressed E-cadherin (Fig. 1C), localized in the cell membrane of the epithelial cells (Fig. 1D). This membranal localization of E-cadherin was not observed on day 10 (Fig. 1B versus 1D), presumably due to its localization within the cytosol. Only very low expression of E-cadherin was detected in the endometrium tissue from RIF patients on day 21 of their normal menstrual cycle (Fig. 1E and F).

Western blot (WB) analysis of endometrial biopsies of fertile women revealed that E-cadherin protein expression increased from the proliferative phase on day 10 (*n* = 9) to the secretory phase on day 21 (*n* = 10) of the menstrual cycle (Fig. 1G and H, *p* < 0.05). Moreover, E-cadherin protein was either absent or hardly expressed in the secretory endometrium of RIF patients (*n* = 12, Fig. 1G and H, *p* < 0.05).

### Long-term endometrial cell viability in the 3D in vitro model

**Fig. 2:**
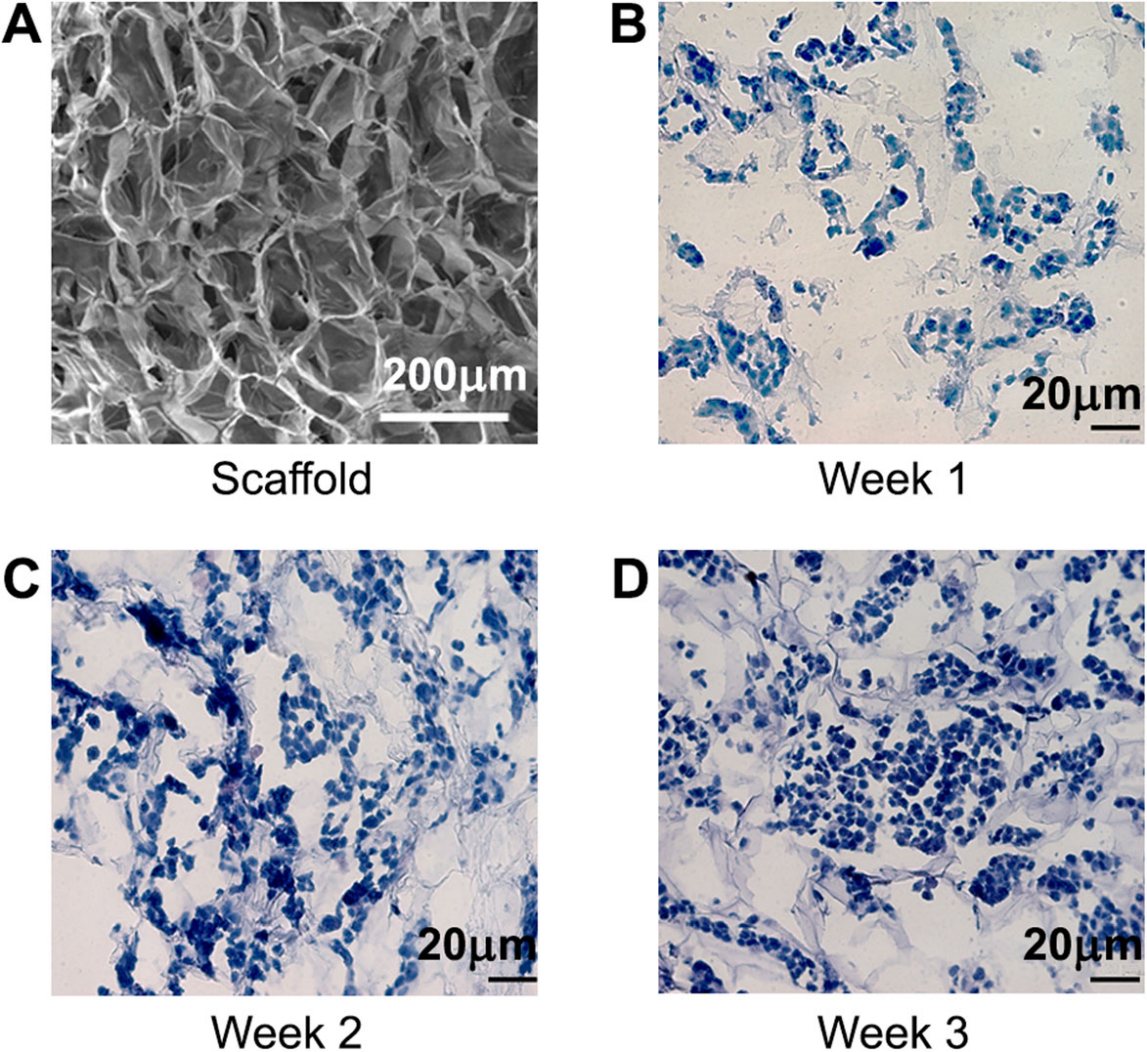
Three-week culture of endometrial cells within macroporous alginate scaffolds. **A** Macroporous structure of alginate scaffold visualized by SEM (Bar: 200 μm). **B-D** H&E staining of thin cryo-sections (10 μm) of 3D endometrial RL95–2 cell constructs within macroporous alginate scaffolds after **B** 1 week, **C** 2 weeks and **D** 3 weeks of cultivation (Bar: 20 μm)

Macroporous alginate scaffolds, fabricated by a freeze-drying technique, had an internal structure of high porosity (> 90%) and interconnecting pores with an average pore size of 80.8 μm and SD of 25 μm (Fig. 2A), similar to previous studies [32], which enabled cell infiltration, accommodation of a large number of cells, and good exposure to nutrients and hormonal treatment.

RL95–2 endometrial epithelial cells (hematoxylin and eosin (H&E) stained) were nested within the interconnected pores of the scaffold; in Fig. 2B – D the infrastructure of the scaffold was evident in grey and no indication of fragmented nuclei was observed. Under static conditions, the cells resided at the surface of the scaffold enabling direct contact with the spheroids. Cell viability was confirmed by MTT tetrazolium salt assay that indicated cell viability for at least 4 weeks (data not shown) and Presto blue (PB) quantitative analysis (Supplementary Fig. 1).

### Hormonal response in the 3D model

**Fig. 3:**
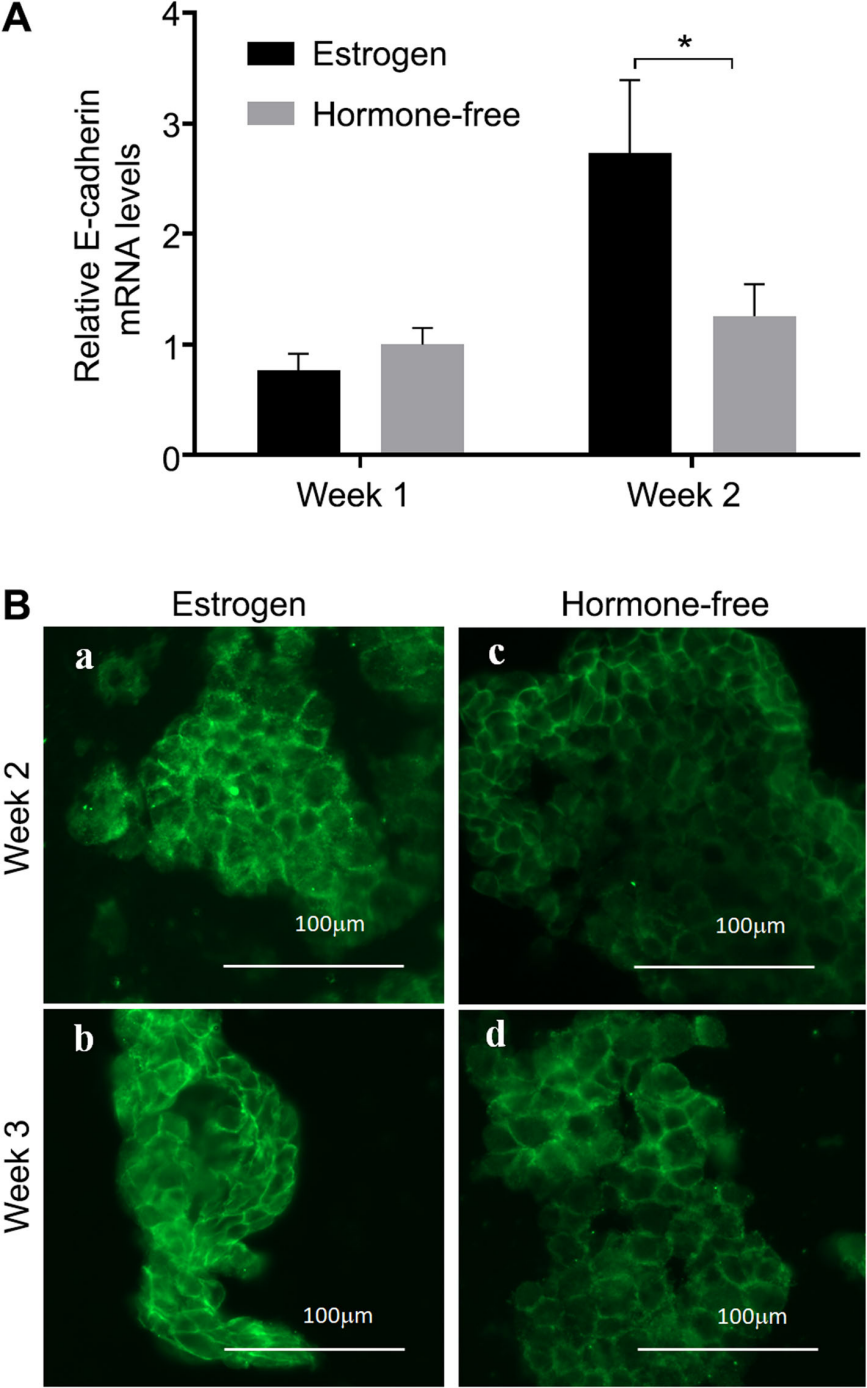
E-cadherin expression in 3D RL95–2 epithelial model after 2 weeks of culture in estrogen-containing medium. **A** Quantification of E-cadherin mRNA expression levels evaluated by quantitative polymerase chain reaction (qPCR). mRNA expression levels were normalized to the ribosomal protein large P0 (RPLP0) mRNA and to expression in 1-week old cell constructs in hormone-free medium (*- *p* < 0.05, 2-way ANOVA, Bonferroni’s post-hoc test, *n* = 8). **B** Representative E-cadherin immunostaining of cryo-sections (10 μm thick) of RL95–2 endometrial cell constructs cultured in (**a**, **b**) estrogen-containing medium or in (**c**, **d**) hormone-free medium for (**a**, **c**) 2 weeks or (**b**, **d**) 3 weeks (Bar: 100 μm).

The mRNA expression levels of E-cadherin in the 3D RL95–2 endometrial model were elevated after 2 weeks of treatment with estrogen-containing medium, compared to hormone-free treatment, confirming model responsiveness to estrogen (Fig. 3A, *p* < 0.05). Moreover, E-cadherin immunostaining indicated that protein expression was more pronounced after 2 (Fig. 3Ba) and 3 (Fig. 3Bb) weeks of estrogen treatment compared to hormone-free treatment at the same time points (Fig. 3Bc and Bd, respectively); further indicating the responsiveness of the model to estrogen. Monolayer, 2D cultures of RL95–2 cells did not survive more than 3 days under hormonal treatment (data not shown).

### Adhesion of JAR in the RL95–2 3D model

**Fig. 4:**
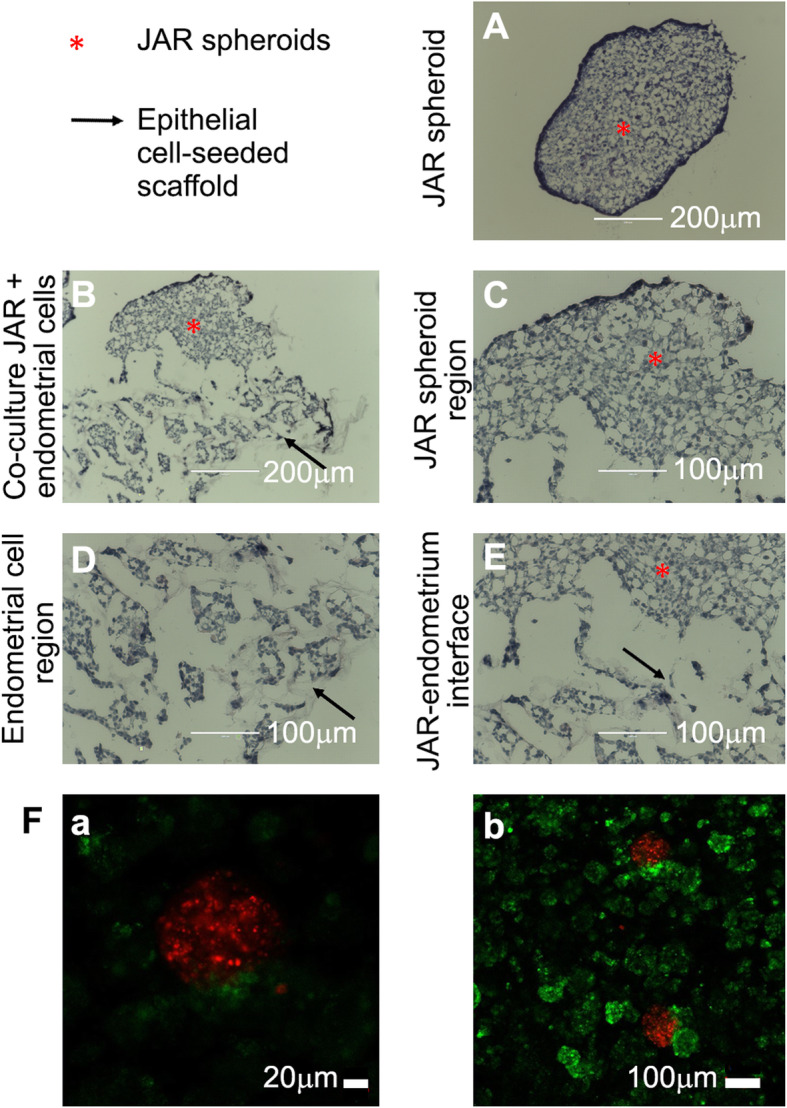
JAR spheroid attachment to 3D 3-week RL95–2 epithelial endometrial model in alginate scaffolds. Co-cultures were incubated for 24 h, and cryo-sections (10 μm thick) were stained by H&E. Star (*) denotes JAR spheroids, arrows are directed at the epithelial cell-seeded scaffold. Representative JAR spheroid (**A**) cultured alone and (**B**) attached to epithelial culture. High magnification of (**C**) JAR spheroid, (**D**) epithelial cells within the macroporous alginate scaffold and (**E**) the interface between the two cell types. (Bar: **A-B**: 200 μm; **C-E**: 100 μm, *n* = 5). **F** Co-culture of red-labeled JAR spheroid and green-labeled RL95–2 cell constructs within alginate scaffolds, after 24 h incubation. (Bar: **a**: 20 μm, **b**: 100 μm)

JAR cells within the spheroid exhibited compact cell morphology with enhanced cell-cell interactions and an overall dense spheroid structure with defined boundaries, as judged by H&E staining (Fig. 4A). JAR spheroids successfully attached to the epithelial endometrial cells when co-cultured with RL95–2 cell constructs (Fig. 4B). The structure of the co-cultured-JAR spheroid was characterized by two areas, one with distinct boundaries, similar to the single spheroid (upper part of the spheroid, Fig. 4B), and the other at the interface between the spheroid and the epithelial cells underneath which showed attachment points (lower part of the spheroid, Fig. 4B, and at higher magnification, Fig. 4C). The epithelial cells were detected within the interconnected pores of the scaffold (Fig. 4D), as shown in Fig. 2B-D, and therefore were easily distinguishable from the JAR cells. The cellular interface between the RL95–2 cells and the JAR spheroids are shown in Fig. 4E. The adhesions of JAR spheroids were observed in all five of the five different RL95–2 cell constructs. Empty scaffolds were incubated with JAR spheroids under the same conditions and no spheroids were observed in any of the scaffolds (data not shown). Non-specific fluorescent membrane staining of RL95–2 cells with PKH67 (green) and JAR spheroids with PKH26 (red), followed by JAR seeding onto the RL95–2-seeded scaffold, confirmed the presence of the two cell types after 24 h of co-culture incubation (Fig. 4Fa and b).

### Restoring estrogen responsiveness in ERα-negative cells in the 3D endometrium-like model

**Fig. 5:**
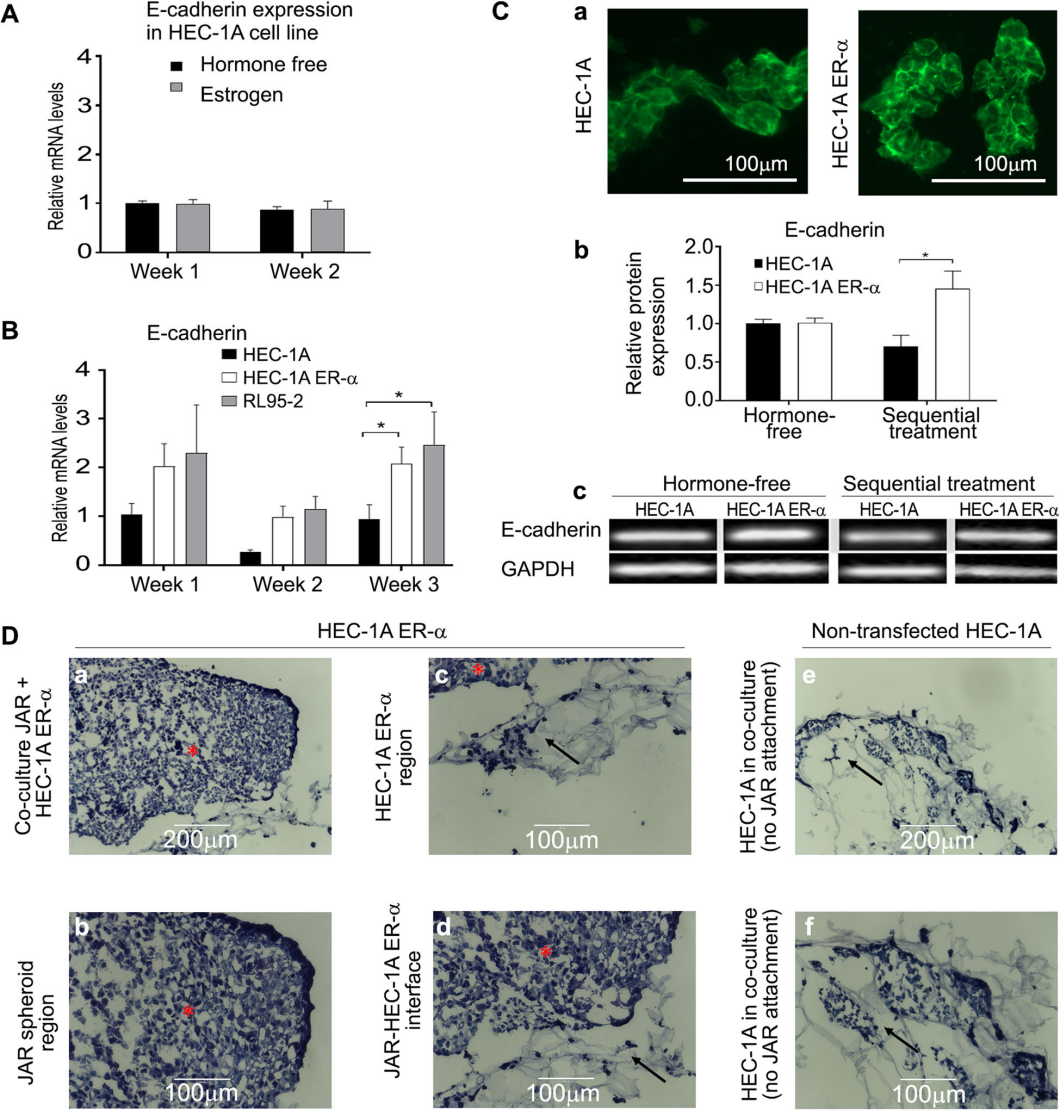
Effect of ERα overexpression on E-cadherin expression and on JAR attachment in ERα-negative cells, cultured with sequential hormonal treatment. **A** E-cadherin mRNA expression levels in estrogen-treated HEC-1A cell constructs after 1 and 2 weeks of culture. Quantification of E-cadherin mRNA expression levels was done by qPCR. mRNA expression levels were normalized to the RPLP0 mRNA and to expression in 1-week old cell constructs in hormone-free medium (*n* = 10). **B** E-cadherin mRNA expression levels in the 3 different culture models were measured by qPCR. The fold change of mRNA levels are relative to constitutively expressed RPLP0 mRNA, and all data sets were normalized to 1-week HEC-1A constructs treated with hormone-free medium (*- *p* < 0.05, 2-way ANOVA, Bonferroni’s post-hoc test, *n* = 12 (HEC-1A), *n* = 11 (HEC-1A-ERα), *n* = 9 (RL95–2)). **C** E-cadherin protein expression. **a** Representative E-cadherin immuno-staining of thin cryo-sections of 3-week-old non-transfected HEC-1A (left) and ERα-transfected HEC-1A (right) cell constructs. (Bar: 100 μm). **b** Quantification of E-cadherin protein expression levels by WB. Expression levels were quantified by the band intensity relative to GAPDH and all data sets were normalized to HEC-1A constructs treated with hormone-free medium (*- *p* < 0.05, 2-way ANOVA, Bonferroni’s post-hoc test, *n* = 3). **c** Representative E-cadherin WB. **D** JAR spheroid attachment to ERα-transfected HEC-1A cell constructs after 3 weeks. Spheroids were observed on 2 of the 3 transfected cell constructs. Representative photomicrographs of (**a**) JAR spheroid attached to ERα-transfected HEC-1A epithelial culture. High magnification of (**b**) a JAR spheroid, (**c**) ERα-transfected HEC-1A epithelial cells within the macroporous alginate scaffolds, and (**d**) the spheroid-epithelial interface. **e** No JAR was attached to any of the three non-transfected HEC-1A cell constructs. **f** High magnification of non-transfected HEC-1A cells within the porous scaffold, with no JAR attachment. (Bar: **a**, **e**: 200 μm, **b-d**, **f**: 100 μm)

HEC-1A cells were used as a model for epithelial cells of RIF non-receptive endometrium. HEC-1A cells, known to express low levels of ERα [33], did not show any increase in E-cadherin expression in response to estrogen treatment (Fig. 5A). HEC-1A were transfected with full-length ERα expressing plasmid. The stable transfection was confirmed by qPCR analysis that indicated elevated mRNA levels of ERα in the transfected cells, compared to cells transfected with an empty vector (Supplementary Fig. 2A, *p* < 0.05). ERα was localized predominantly in the nuclei, as seen by specific immunofluorescent staining for ERα (Supplementary Fig. 2B).

Transfected cells were cultured within macroporous alginate scaffolds for 3 weeks under sequential hormonal treatment. These exhibited significantly higher E-cadherin mRNA expression levels compared to those of HEC-1A constructs (Fig. 5B, *p* < 0.05). The expression levels of the transfected cells were slightly lower but not significantly different from those of RL95–2 cell constructs (Fig. 5B).

Immuno-staining for E-cadherin in the cell constructs revealed pronounced protein expression at cell boundaries of ERα-transfected HEC-1A cells in comparison to the non-transfected HEC-1A cells where slightly lower protein expression was observed (Fig. 5Ca).

WB analysis indicated that E-cadherin protein expression levels in constructs of ERα-transfected HEC-1A cells cultured with sequential hormonal treatment, were over two-fold higher after 2 weeks relative to non-transfected HEC-1A cells (Fig. 5Cb and c, *p* < 0.05).

JAR spheroids were observed on 2 of 3 transfected cell constructs (Fig. 5Da-d). When non-transfected HEC-1A cell constructs were used, no JAR attachment was observed to any of the three HEC-1A cell constructs (Fig. 5De-f), despite the cell population having the same gross morphology as in the RL95–2 cell constructs.

## Discussion

This study presents a novel 3D model for epithelial-embryo interactions in 3D macroporous alginate scaffolds. The endometrial epithelial cells that were cultured within the porous structure formed a tissue-like structure that was viable for 3 weeks under hormone treatment that mimicked the entire typical menstrual cycle. The newly-formed epithelial tissue-like construct of the receptive RL95–2 cell line was responsive to hormones, as judged from the hormone-dependent E-cadherin expression levels; and, most importantly, the RL95–2 cell constructs were functional in terms of their ability to adhere to JAR spheroids, mimicking the first step of implantation in fertile women.

A second 3D model was established that partially mimics the endometrium of RIF patients with non-receptive endometrium tissue. To this end, HEC-1A epithelial cells, expressing low levels of ERα [33], were cultured within alginate scaffolds under the same conditions as the receptive RL95–2 constructs. The HEC-1A 3D model demonstrated lower levels of E-cadherin and JAR spheroids could not attach to the cell constructs, as opposed to the receptive RL95–2 3D model. ERα overexpression in HEC-1A cells restored E-cadherin expression both at the mRNA and protein levels. JAR spheroid attachment capability was also recovered in the ERα-transfected cells, probably due to the enhanced adhesion mediated by E-cadherin, facilitating the cell-cell interactions with the embryo model [11]. Collectively, both models emphasize the importance of ERα and E-cadherin in the initial steps of embryo attachment and indicate a possible regulation mechanism of E-cadherin by ERα [34, 35].

The presented in vitro 3D models showed lower expression of E-cadherin in the RIF model of HEC-1A cells compared to the receptive RL95–2, in agreement with analyses of endometrium tissue of fertile women compared to that of RIF patients [6]. These clinical data together with the in vitro model results support previous reports demonstrating significantly lower levels of ERα and higher levels of the E-cadherin repressor, Slug transcription factor [36], in RIF endometrium [6].

The presented 3D RL95–2 model expressed ERα, was responsive to hormones, expressed E-cadherin and enabled JAR attachment, making it a good in vitro model for fertile endometrium based on the clinical results presented here and previously [6]. The 3D HEC-1A model with lower levels of ERα, expressed lower levels of E-cadherin and could not facilitate JAR attachment, making it an in vitro model for RIF endometrium.

The proposed novel 3D model based on macroporous alginate scaffolds represents a significant advantage in its ability to support a long-term viable culture of endometrial cells for several weeks under hormonal treatment, with observed hormone responsiveness and temporal tissue functionality, as demonstrated by JAR attachment. The 3D highly porous alginate scaffold, with interconnected pores, enabled extended viability presumably due to better mass transfer and accessibility to nutrients and hormones from the culture medium as well as to essential paracrine factors. The porous scaffold structure, with pore sizes of ~ 80 μm, favors cell-cell and cell-matrix interactions with the secreted extracellular matrix (ECM), allowing better organization into tissue-like structures and functionality. Other 3D systems utilizing hydrogel networks are highly dense precluding efficient mass transfer, long-term viability and cell organization into tissue structure.

While alginate biomaterial simulates the hydrated structure of native ECM [37], it is cell-inert as no specific interactions are formed to the alginate scaffold [38]. This provides a means to examine cell organization without any biochemical effects of the biomaterial itself.

The developed in vitro 3D implantation model may serve as a platform for further studying the RIF endometrium and to ultimately increase endometrial receptivity. Such a model is of significant potential since the current evaluation of IVF treatment failure is mostly limited to analyzing the endometrium thickness [39].

Whereas this study addressed the initial step of blastocyst attachment to the epithelial cell layer, future studies using these models may utilize primary epithelial and stromal cells from the endometrium of RIF patients to examine individual failure mechanisms and to design therapies targeted at all stages of the embryo implantation process.

## Conclusions

Taken together, the proposed novel 3D models of endometrium cells within macroporous alginate scaffolds enabled long-term culture of viable human endometrial cells under hormone stimulation that mimics the normal menstrual cycle. This model provides a platform for elaborate studies of the regulatory mechanisms involved in the implantation process and can set the basis for a broader tool for designing and testing novel therapeutic strategies for recurrent implantation failure.

## Materials and methods

### RIF and fertile patients

The clinical study was performed as previously described [6]. The study group consisted of patients under the age of 38 years with RIF, from the Hadassah University Hospital, IVF unit. Only patients who had undergone at least three IVF-embryo transfer failures, in which no less than 10 high quality embryos were transferred in total, were included. The control group of fertile women consisted of volunteer women under the age of 40, who had at least one normal pregnancy and delivery. We excluded women who had a past record of infertility, those currently on oral contraceptive therapy and those with intrauterine contraceptive devices.

All patients had a good hormonal reserve (FSH < 8 mIU/mL) and a good response to hormonal stimulation (more than eight oocytes/oocyte retrieval). Patients were confirmed to have a normal uterine cavity by office hysteroscopy and normal endometrial thickness. Participants were instructed not to use hormone therapy during the research period.

All participants signed informed consent forms following the approval of the institutional ethics committee (number 14–11/10/02).

### Endometrial sampling

Endometrial biopsies were collected using a Pipelle® de Cornier device (CCD Laboratories, Paris, France) on day 10 and day 21 of the menstrual cycle, as previously described [6]. Endometrial samples of day 21 from patients and controls were confirmed to be postovulatory when serum progesterone level was above 20 ng/mL. Samples were immediately transferred to the laboratory and processed within 1 h.

### Macroporous alginate scaffold fabrication

Alginate scaffolds with a diameter of 5 mm and 2 mm thickness were fabricated from low viscosity (LVG) alginate (LVG with MW 100 kDa, > 65% guluronic acid monomer content, NovaMatrix FMC Biopolymers, Drammen, Norway) by a freeze-drying technique as previously described [29]. In short: Alginate was dissolved in double distilled water (DDW,1.2% (w/v) solution) and then was cross-linked with calcium by adding a solution of D-gluconic acid hemicalcium salt (1.2% w/v) under homogenization for 2 min. Final component concentrations in the cross-linked solution was 1.0 and 0.2% (w/v) in DDW for the polymer and cross-linker, respectively. The cross-linked solution was poured into 96-well plates (100 μL/well), frozen at − 20 °C for 24 h and then lyophilized. The scaffolds were sterilized by exposure to ultra-violet light in a biological hood for 1 h.

Pore size in the scaffolds was measured by scanning electron microscope (SEM, Tescan, VEGA3) imaging of dry scaffolds.

### Cell construct preparation and cultivation

RL95–2 (CRL-1671; American Type Culture Collection, Frederick, MD) was derived from a moderately differentiated adenosquamous carcinoma of the endometrium and was used as a model for receptive endometrium [7]. HEC-1A (HTB-112 American Type Culture Collection, Frederick, MD) was derived from human endometrial carcinoma and served as a model of the nonreceptive state [7]. The human choriocarcinoma cell line JAR (HTB-144 American Type Culture Collection, Frederick, MD), cultured to form multicellular spheroids, was used as a model of blastocysts [7].

#### Preparation for 3D culture

RL95–2 and JAR cells were grown in high-glucose Dulbecco’s modified Eagle’s medium (DMEM) and HEC-1A cells were cultured in McCoy’s medium. Both media were supplemented with 10% fetal calf serum (FCS), 1% penicillin-streptomycin-amphotericin solution, 1% L-glutamine, 1% sodium pyruvate, 1% non-essential amino acids and 1% vitamins (all v/v). The medium was replaced twice a week until cells reached 80% confluence, at which stage they were seeded onto the scaffolds.

#### 3D culture

Cell were seeded onto the scaffolds by applying 15 μL of the cell suspension onto the dry scaffold (3–5 × 10^5^ cells/scaffold) followed by centrifugation (100×g, 2 min) and incubation for 0.5 h at 37 °C under 5% CO_2_. Subsequently, seeded scaffolds were transferred to 750 μL of culture medium at 37 °C under 5% CO_2_. The regular menstrual cycle was modeled by cell constructs that were cultured for 3 weeks under sequential hormonal treatment of one-week estrogen priming (10^− 8^ M), followed by 2 weeks of progesterone treatment (10^− 7^ M), or in control media: (a) hormone-free, (b) estrogen (10^− 8^ M). The medium was replaced twice a week.

### RNA isolation and quantitative polymerase chain reaction (qPCR)

Cell constructs were incubated with 0.5 mL of Tri-Reagent commercial kit (Sigma-Aldrich) according to the manufacturer’s instructions. The extracted RNA was converted to cDNA with a qScript cDNA synthesis kit (Quanta Biosciences, Gaithersburg, MD).

Oligonucleotide primers were designed by the Primer Express program (Applied Biosystems, Foster City, CA, USA) and were as follows:

- E-cadherin: sense 5′-gccatcgcttacaccatcct-3′, antisense 5′-ggcacctgacccttgtacgt-3′.
- ERα: sense 5′-cggcattctacaggccaaa-3′, antisense 5′-gcgagtctccttggcagattc-3′.
- RPLP0 housekeeping gene [40]: sense 5′-ccaactacttccttaagatcatccaacta-3′, antisense 5′-acatgcggatctgctgca-3′.

qPCR was performed with a KAPA SYBRFAST Universal qPCR kit (Kapa Biosystems, Wilmington MA) on the CFX Connect Real-Time system (Bio-Rad Laboratories, Hercules CA). Analysis was performed with BioRad CFX Manager Software. Expression levels in each sample were normalized to RPLP0 levels.

### Western blotting (WB)

Endometrial tissue samples as well as in vitro cells constructs were lysed in a lysis buffer (1% NP40, 20 mM Tris–HCl (pH 7.5), 137 mM NaCl, 0.5 mM Ethylenediaminetetraacetic acid

Ethylenediaminetetraacetic acid (EDTA), 10% glycerol, 1 mM sodium orthovanadate, 1X protease inhibitors cocktail and 0.1% sodium dodecyl sulfate (SDS), (Sigma, St. Louis, MO, USA)). About 10 μg of protein from each sample were loaded under reducing conditions on 10% SDS-polyacrylamide gels, separated by electrophoresis, transferred to polyvinylidene fluoride (PVDF) membranes. (Millipore, Bedford, MA, USA), blocked in Tris buffered saline with Tween 20 (TBST; 10 mM Tris–HCl (pH 8.0), 150 mM NaCl, and 0.1% Tween 20) containing 5% non-fat dry milk (Nestle, Vevey, Switzerland) for 1 h at room temperature. Blots were then incubated overnight at 4 °C with either (a) primary mouse antibody against human E-cadherin (HECD-1 clone, Alexis Biochemicals, Lausen, Switzerland) or (b) anti-tubulin antibody (Sigma), or (c) primary rabbit antibody against GAPDH (14C10, Cell Signaling, Danvers, MA); followed by 1 h incubation with anti-mouse or anti-rabbit HRP-conjugated secondary antibodies, respectively (Jackson Immuno-Research Laboratories, Inc., West Grove, PA). Enhanced chemiluminescence (ECL) procedure was performed using an ECL-EZ kit (Biological Industries) with signal detection by Image-Lab (Bio-Rad Laboratories Ltd., UK). Protein bands were quantified using ImageJ software (NIH) and normalized to GAPDH expression levels.

### Histology and immunostaining

Endometrial biopsy samples were embedded in paraffin for histological evaluation. Paraffin sections were immuno-stained for human E-cadherin with E-cadherin primary antibody.

In vitro cell constructs were embedded in optimal cutting temperature compound (OCT) (Sakura, NL) and were frozen at − 20 °C for 2 h, then transferred to − 80 °C until sectioning. Frozen constructs were cut into 10 μm thick sections by cryostat, mounted on slides, and stained for hematoxylin and eosin (H&E). For immunofluorescence, the cryo-sections were dried at room temperature (RT) and dehydrated in DMEM-based buffer (CaCl_2_‧2H_2_O 0.265 g/L, KCl 0.400 g/L, MgSO_4_‧7H_2_O 0.200 g/L, NaCl 6.400 g/L, NaHCO_3_ 3.700 g/L, NaH_2_PO_4_ 0.109 g/L, pH 7.2–7.4). Subsequently, sections were fixed in 4% neutral-buffered formalin for 10 min at RT, washed and blocked for 1 h at RT in DMEM buffer containing 3% BSA (w/v). Cryo-sections were incubated with E-cadherin primary antibody at 4 °C overnight and washed and incubated for 0.5 h with Alexa Fluor 488-conjugated goat anti-mouse secondary antibody. Finally, the sections were washed and mounted on slides. Samples were examined by a Cytation™ 3 microscope.

### JAR spheroid attachment to 3D endometrial model

JAR trophoblast spheroids were prepared as indicated with some modifications [41]. Briefly, JAR cells were transferred to 6-well plates (2.5 × 10^5^ per well) and agitated at 37 °C on a shaker for 24 h. Then, the formed spheroids were collected and supernatant removed. A volume of 6 μL of the suspended spheroids were seeded on top of 3-week old endometrial cell constructs within alginate scaffolds. The co-cultures were incubated for 24 h to allow spheroid attachment and then embedded in OCT and frozen. Cryo-sections of the co-culture were stained by H&E.

### Fluorescent membranal staining

For visualization of the two cell types, red and green fluorescent general membrane cell linkers were used: PKH26 red fluorescent staining for JAR spheroid and PKH67 green fluorescent staining for RL95–2 cell-seeded scaffolds. Staining with PKH26 was done by incubating the JAR culture suspension with the dye as instructed by the manufacturer prior to spheroid formation. For PKH67, the provided manufacturer’s protocol needed to be adapted for 3D culture. In brief, seeded scaffolds were first washed with serum-free medium (3 times), then incubated with PKH67 for 5 min and stopped with 1 min incubation with FCS, followed by 3 washing steps with culture medium. Immediately after staining, the red-labeled JAR spheroids were seeded onto the green-labeled RL95–2 3D cell constructs within the alginate scaffold, and the co-culture was incubated for 24 h. The co-culture was visualized by confocal microscopy ZEISS LSM710.

### Statistical analysis

Statistical analysis was performed with GraphPad Prism, version 6.01 for Windows (GraphPad Software, San Diego, CA). All variables were expressed as mean ± SEM.

E-cadherin protein expression of human samples were compared by 1-way analysis of variance (ANOVA). Dunnett’s post-hoc test was carried out to determine differences. E-cadherin mRNA and protein of in vitro models were compared by 2-way repeated measures ANOVA. Bonferroni’s post-hoc test was carried out to determine differences. *p* < 0.05 was considered to be statistically significant.

## Supplementary information

**Additional file 1: Supplementary data 1.** Presto blue (PB) quantitative analysis of RL95–2 cell constructs. RL95–2 cell-seeded scaffolds were incubated for 2 h with 10% (v/v) PB reagent (in DMEM medium, supplemented with 10% (v/v) FCS). Then samples of 100 μL of the medium were transferred to a black bottom 96-well plate and fluorescent readings were obtained at excitation and emission wavelengths of 560 nm and 590 nm, respectively. A calibration curve was prepared to quantify viable cells. Cell numbers at each time point were calculated by using a calibration curve and were normalized to the number of cells seeded into the scaffolds. **Supplementary Fig. 1.** (A) RL95–2 cell viability after 1- and 2-weeks culture, by PB analysis. PB analysis of scaffolds, seeded with 0.5×10^6^ cells showed no significant decrease throughout 2 weeks culture. (B) Analysis of scaffolds, seeded with 6.25 × 10^4^ cells, one order of magnitude less than A, showed a steady cell number after 1 week of culture and a significant increase after 2 weeks of culture.

**Additional file 2: Supplementary data 2.** ERα transfection of HEC-1A cells. The ERα open reading frame was cloned into a pcDNA6.2/V5 vector (a kind gift from Prof. Carlos Simon, University of Valencia). HEC-1A cells were transfected using Lipofectamine™ 2000 (Invitrogen, Paisley, UK) either with the ERα vector or with an empty vector as control. Following 48 h, medium was replaced with 10 μg/mL blasticidin-containing media (Invitrogen, Paisley, UK) for selection. After 2 weeks, individual colonies were selected. Transfection efficiency was confirmed by ERα mRNA expression levels, evaluated by qPCR and ERα nuclear localization, and evaluated by immunofluorescent staining. **Supplementary Fig. 2.** Validation of ERα transfection evaluated by ERα expression in ERα transfected HEC-1A cells compared to HEC-1A cells transfected with the empty vector. **(A) qPCR analysis:** Higher ERα mRNA expression levels in ERα transfected HEC-1A cells, compared to cells transfected with an empty vector (t-test, *p* < 0.01). Expression levels are relative to RPLP0 levels. **(B) Anti-ERα immunofluorescent staining:** ERα protein expression in ERα transfected HEC-1A cells (right), compared to cells transfected with an empty vector (left). ERα immunofluorescent staining (green) and 4′,6-diamidino-2-phenylindole (DAPI) staining for nuclei (blue) (Bar: 100 μm).

## Data Availability

The datasets during and/or analyzed during the current study available from the corresponding author on reasonable request.

## Abbreviations

ANOVA: Analysis of variance
DDW: Double distilled water
DMEM: Dulbecco’s modified Eagle’s medium
ECL: Enhanced chemiluminescence
EDTA: Ethylenediaminetetraacetic acid
ERα: Estrogen receptor alpha
FCS: Fetal calf serum
GAPDH: Glyceraldehyde 3-phosphate dehydrogenase
H&E: Hematoxylin and Eosin
OCT: Optimal cutting temperature
PB: Presto blue
qPCR: Quantitative polymerase chain reaction
RIF: Recurrent implantation failure
RPLP0: Ribosomal protein lateral stalk subunit P0
RT: Room temperature
SDS: Sodium dodecyl sulfate
TBST: Tris buffered saline with Tween 20
WB: Western blot
WOI: Window of implantation
