# Supplementary figures and images for: Novel 3D embryo implantation model within macroporous alginate scaffolds
Source: J Biol Eng. 2020 Jun 30;14:18. doi: 10.1186/s13036-020-00240-7 (PMC7325373; doi:10.1186/s13036-020-00240-7)

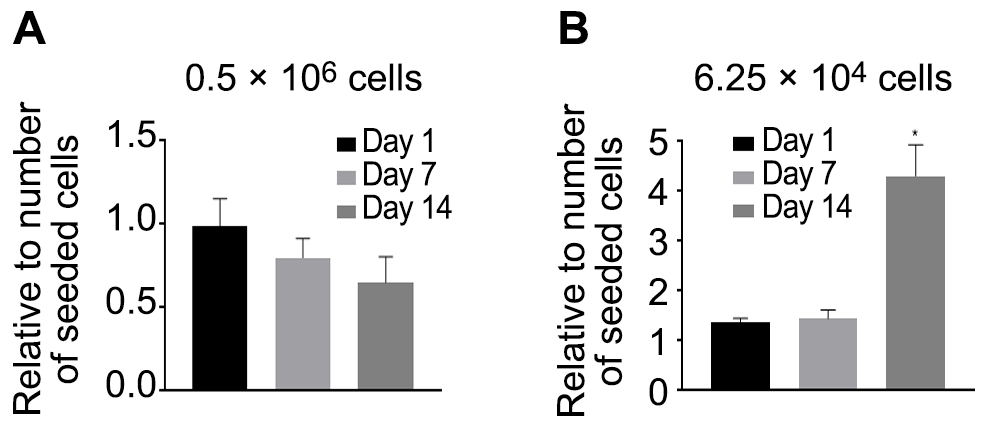

Supplement: Supplementary file 1 — Additional file 1: Supplementary data 1. Presto blue (PB) quantitative analysis of RL95–2 cell constructs. RL95–2 cell-seeded scaffolds were incubated for 2 h with 10% (v/v) PB reagent (in DMEM medium, supplemented with 10% (v/v) FCS). Then samples of 100 μL of the medium were transferred to a black bottom 96-well plate and fluorescent readings were obtained at excitation and emission wavelengths of 560 nm and 590 nm, respectively. A calibration curve was prepared to quantify viable cells. Cell numbers at each time point were calculated by using a calibration curve and were normalized to the number of cells seeded into the scaffolds. Supplementary Fig. 1. (A) RL95–2 cell viability after 1- and 2-weeks culture, by PB analysis. PB analysis of scaffolds, seeded with 0.5×10^6^ cells showed no significant decrease throughout 2 weeks culture. (B) Analysis of scaffolds, seeded with 6.25 × 10^4^ cells, one order of magnitude less than A, showed a steady cell number after 1 week of culture and a significant increase after 2 weeks of culture. [file 13036_2020_240_MOESM1_ESM.tif]

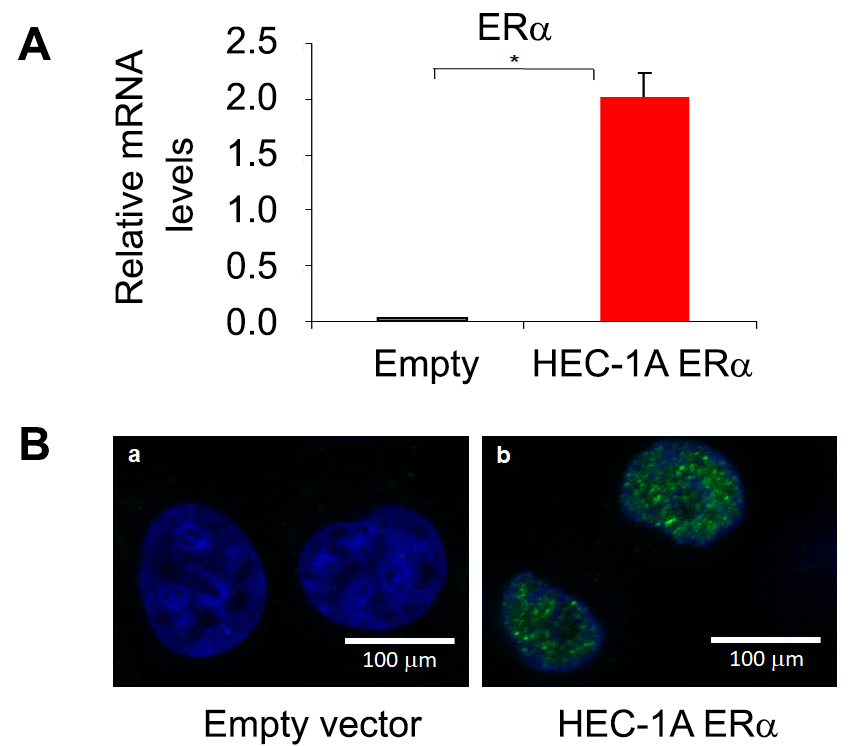

Supplement: Supplementary file 2 — Additional file 2: Supplementary data 2. ERα transfection of HEC-1A cells. The ERα open reading frame was cloned into a pcDNA6.2/V5 vector (a kind gift from Prof. Carlos Simon, University of Valencia). HEC-1A cells were transfected using Lipofectamine™ 2000 (Invitrogen, Paisley, UK) either with the ERα vector or with an empty vector as control. Following 48 h, medium was replaced with 10 μg/mL blasticidin-containing media (Invitrogen, Paisley, UK) for selection. After 2 weeks, individual colonies were selected. Transfection efficiency was confirmed by ERα mRNA expression levels, evaluated by qPCR and ERα nuclear localization, and evaluated by immunofluorescent staining. Supplementary Fig. 2. Validation of ERα transfection evaluated by ERα expression in ERα transfected HEC-1A cells compared to HEC-1A cells transfected with the empty vector. (A) qPCR analysis: Higher ERα mRNA expression levels in ERα transfected HEC-1A cells, compared to cells transfected with an empty vector (t-test, p < 0.01). Expression levels are relative to RPLP0 levels. (B) Anti-ERα immunofluorescent staining: ERα protein expression in ERα transfected HEC-1A cells (right), compared to cells transfected with an empty vector (left). ERα immunofluorescent staining (green) and 4′,6-diamidino-2-phenylindole (DAPI) staining for nuclei (blue) (Bar: 100 μm). [file 13036_2020_240_MOESM2_ESM.tif]
